# Comparison of Remote Sensing Methods for Plant Heights in Agricultural Fields Using Unmanned Aerial Vehicle-Based Structure From Motion

**DOI:** 10.3389/fpls.2022.886804

**Published:** 2022-06-24

**Authors:** Ryo Fujiwara, Tomohiro Kikawada, Hisashi Sato, Yukio Akiyama

**Affiliations:** Hokkaido Agricultural Research Center, National Agriculture and Food Research Organization (NARO), Sapporo, Japan

**Keywords:** unmanned aerial vehicle, structure from motion, remote sensing, plant height, 3D structure analysis, maize

## Abstract

Remote sensing using unmanned aerial vehicles (UAVs) and structure from motion (SfM) is useful for the sustainable and cost-effective management of agricultural fields. Ground control points (GCPs) are typically used for the high-precision monitoring of plant height (PH). Additionally, a secondary UAV flight is necessary when off-season images are processed to obtain the ground altitude (GA). In this study, four variables, namely, camera angles, real-time kinematic (RTK), GCPs, and methods for GA, were compared with the predictive performance of maize PH. Linear regression models for PH prediction were validated using training data from different targets on different flights (“different-targets-and-different-flight” cross-validation). PH prediction using UAV-SfM at a camera angle of –60° with RTK, GCPs, and GA obtained from an off-season flight scored a high coefficient of determination and a low mean absolute error (MAE) for validation data (*R*^2^*_*val*_* = 0.766, MAE = 0.039 m in the vegetative stage; *R*^2^*_*val*_* = 0.803, MAE = 0.063 m in the reproductive stage). The low-cost case (LC) method, conducted at a camera angle of –60° without RTK, GCPs, or an extra off-season flight, achieved comparable predictive performance (*R*^2^*_*val*_* = 0.794, MAE = 0.036 m in the vegetative stage; *R*^2^*_*val*_* = 0.749, MAE = 0.072 m in the reproductive stage), suggesting that this method can achieve low-cost and high-precision PH monitoring.

## Introduction

Remote sensing is a key technology for the sustainable management of agricultural fields. Agricultural management based on remote sensing strengthens food production and reduces natural resource use. Thus, remote sensing technologies have found applications, such as growth monitoring, irrigation management, weed detection, and yield prediction (Sishodia et al., 2020). Furthermore, the applications of remote sensing in agriculture have gained widespread attention in recent years (Weiss et al., 2020).

Unmanned aerial vehicles (UAVs) are commonly used for the remote sensing of agricultural fields owing to their high-resolution imagery and cost-effectiveness. Sensors (e.g., RGB or multispectral cameras, laser scanning devices, etc.) and processing strategies (e.g., vegetation index calculation, machine learning, 3D structure analysis, etc.) have been combined to solve problems in remote sensing applications (Tsouros et al., 2019).

Three-dimensional (3D) structural analysis is useful for determining plant height (PH) and volume, which reflect the growth and biomass of crops (Yao et al., 2019). Strategies for 3D structure analysis include generating 3D models from multiview aerial images of UAVs using structure from motion (SfM) algorithms or obtaining 3D point clouds with light detection and ranging (LiDAR) systems (Paturkar et al., 2021). The SfM approach with UAV imagery (UAV-SfM) can be conducted at a relatively low cost using normal RGB cameras to suit the requirements of agricultural applications. The UAV-SfM approach for monitoring growth or biomass has been applied to various crops, such as wheat (Holman et al., 2016; Madec et al., 2017; Volpato et al., 2021), barley (Bendig et al., 2013, 2014), rice (Jiang et al., 2019; Kawamura et al., 2020; Lu et al., 2022), and maize (Li et al., 2016; Ziliani et al., 2018; Tirado et al., 2020).

However, the UAV-SfM approach has a problem with regard to balancing precision and cost. During the SfM process, matching features over multiple images are detected, camera positions are estimated, and dense point clouds are generated (Westoby et al., 2012). Ground control points (GCPs), which are points whose coordinates are known from ground surveys, are often used to correct the camera positions. The coordinates of each aerial image surveyed with the global navigation satellite system (GNSS) are commonly recorded in the metadata of the image and can be used for the SfM process. SfM analysis without GCPs often faces coordinate errors owing to the uncertainty of GNSS (Wu et al., 2020) or an SfM-specific distortion termed the central “doming” effect (Rosnell and Honkavaara, 2012). However, the installation and maintenance of GCPs on the ground require time and effort. Additionally, annotation of GCPs on aerial images also takes time if weeds or reflection of sunlight interfere with the auto-detection algorithms for GCPs.

An orthomosaic (an orthographic image composed of geometrically corrected aerial images) and a digital surface model (DSM; a representation of elevation on the 3D model) are constructed from the point clouds. When aerial images of an on-season agricultural field are taken and processed, a DSM obtained by the SfM process shows an elevation that includes the PH. To extract PH from the DSM, data for ground altitude (GA), such as that obtained from a digital terrain model (DTM; digital topographic maps indicating ground surface), are necessary.

Ground altitude is mainly obtained by processing off-season (pre-germination or post-harvest) images to create a DTM (Roth and Streit, 2018; Ziliani et al., 2018; Jiang et al., 2019; Kawamura et al., 2020) or by extracting the altitude of the soil surface in the on-season DSM (Tirado et al., 2020). The former method (processing off-season images) requires an extra flight, along with the SfM process. In addition, the models obtained from different flights generally deviate from each other owing to the uncertainty of the coordinates and distortion of the 3D models mentioned above. Such deviations cause errors in PH prediction and affect reproducibility. Therefore, correction with GCPs is crucial for this method. The latter method (extracting soil altitude) requires the presence of a bare soil surface in the seasonal DSM. However, this method may be difficult to apply when the ground is fully covered by plants. To obtain GA in plants, extracted soil coordinates were interpolated to generate a DTM (Murakami et al., 2012; Gillan et al., 2014; Iqbal et al., 2017). Such interpolation methods can achieve low-cost and accurate PH predictions when adequate soil coordinates are extracted for the interpolation algorithm. Although other sources, such as airborne laser scanning (ALS), can be used to determine the altitude of the terrain (Li et al., 2016), the applicable scope is mostly restricted because of the equipment costs and time demands for aerial scanning.

The precision of UAV-SfM analysis is determined by the camera angles, real-time kinematics (RTK), and GCPs. SfM point clouds generated from aerial images taken at diagonal camera angles can have fewer errors that result from the “doming” effect (James and Robson, 2014). Furthermore, UAVs equipped with high-precision positioning systems using RTK-GNSS have become increasingly popular, although the initial and operational costs for RTK-UAVs remain higher. Finally, GCPs are generally used to correct camera positions, as mentioned above. In addition to these three parameters, methods for obtaining GA need to be considered for PH prediction. Although comparative studies have been conducted on one or a few of these variables (Holman et al., 2016; Xie et al., 2021; Lu et al., 2022), their effects on the precision of PH monitoring have not been fully investigated.

**FIGURE 1:**
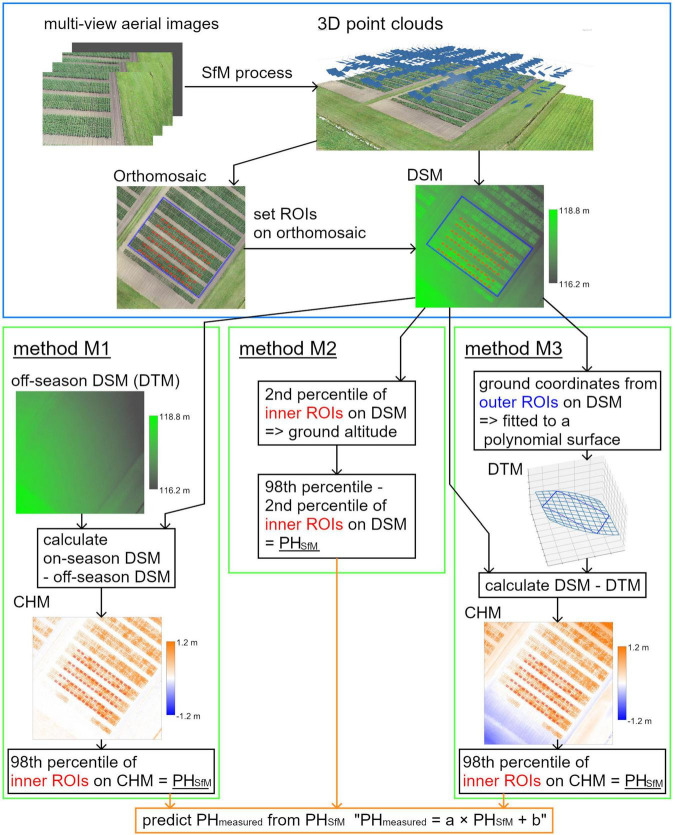
An overview of the SfM process and three methods for obtaining ground altitude (GA); methods M1, M2, and M3. A DSM is a digital surface model that represents elevation on the 3D model, a DTM is a digital terrain model that represents elevation without plants, and a CHM is a crop height model that represents plant height of crop.

In this study, four variables, namely, camera angles, RTK, GCPs, and methods for obtaining GA, were compared for PH prediction in maize. Two existing methods for obtaining GA, using off-season DSMs (method M1) and extracting the altitude of the soil surface (method M2), were demonstrated (Figure 1). In addition, an interpolation method for obtaining GA (method M3) was considered, wherein the coordinates of the terrain around the field were obtained and fitted to a polynomial surface. The surface can then be used as a DTM, and the DTM subtracted from a DSM provides a crop height model (CHM), representing the PH of the crop (Chang et al., 2017). This method can achieve high precision without GCPs, even when the inside of the field is covered with plants.

## Materials and Methods

### Data Collection and Structure From Motion Process

**FIGURE 2:**
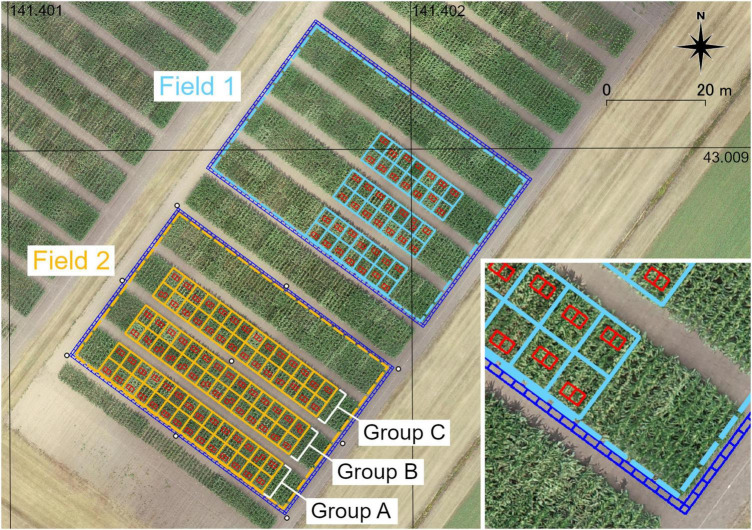
An overview of two fields of maize used in this study. Light blue (Field 1) and orange (Field 2) solid lines show the plots. Dash lines show the analysis region of each field. White circles around or in Field 2 show the locations of GCPs. Red rectangles show ROIs on rows (inner ROIs). Blue rectangles show ROIs around the field (outer ROIs).

The data were collected from the Hokkaido Agricultural Research Center (Hokkaido, Japan). Two maize fields (Fields 1 and 2) under variety tests were used in this study. An overview of these two fields is presented in Figure 2. Field 1 was used for method M3, and Field 2 was used for validation of PH prediction under all conditions of camera angles, RTK, GCPs, and methods for obtaining GA. There were 42 plots (14 varieties) in Field 1 and 84 plots (21 varieties) in Field 2. Each plot had four rows, and each row contained 18 plants. The row spacing was 0.75 m and the in-row plant spacing was 0.18 m (7.41 plants per square meter) in both fields.

Nine checkerboard square markers used as GCPs for the SfM process were placed in Field 2. Eight GCPs were located around the field and one GCP was located at the center of the field. The locations of the GCPs are shown in Figure 2. The coordinates of the GCPs were surveyed using D-RTK 2 (SZ DJI Technology, Nanshan, Shenzhen, China).

The ground truth of PH was measured using rulers as the height from the ground to the highest point of the plant at two different growth stages. During the vegetative stage, the highest point was at the apex of the top leaf, while during the reproductive stage, the highest point was at the apex of the tassel. Actual measurements were conducted in the middle two of the four rows in each plot. The PH of five consecutively placed plants in each row was measured and averaged. The average from one row was regarded as one sample (*PH*_measured_). Subsequently, *PH*_measured_ was obtained from 84 rows in Field 1 and 168 rows in Field 2. The schedule of the actual PH measurements and UAV image acquisition is presented in Table 1.

**TABLE 1:** The schedule of data collection.

Stage	Process	Field 1		Field 2	
	Sowing	2021/5/12		2021/5/12	

Pre- germination	Image acquisition	–		2021/5/14	4 flights (4 conds. × 1 rep.)*
				2021/5/17	8 flights (4 conds. × 2 reps.)*

Vegetative stage	Image acquisition	2021/6/28	3 flights (3 reps.)	2021/6/30	12 flights (4 conds. × 3 reps.)
	Measurement	2021/6/28		2021/6/30	

Reproductive stage	Image acquisition	2021/8/4	3 flights (3 reps.)	2021/9/2	12 flights (4 conds. × 3 reps.)
	Measurement	2021/8/6		2021/9/3	

*Three repetitions of image acquisition in the pre-germination stage (Field 2) were conducted over 2 days: one rep on 2021/5/14, and the remaining two reps on 2021/5/17. The details of UAV image acquisition are shown in Supplementary Table 1 (Field 1) and Supplementary Table 2 (Field 2).

**FIGURE 3:**
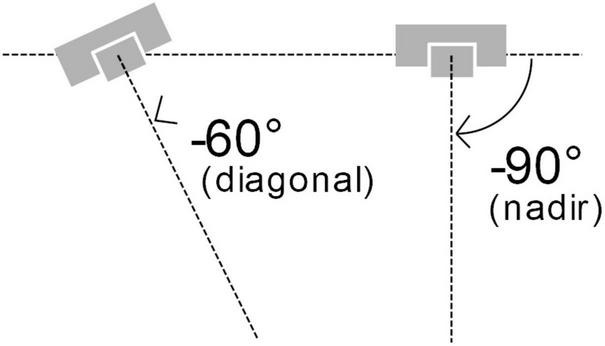
An illustration of the camera angle.

A DJI Phantom 4 RTK (SZ DJI Technology) with a mounted camera (lens: 8.8 mm focal length, sensor: 1” CMOS 20 M) was used for image acquisition. The analysis region (35.5 m × 54 m) for the SfM process in each field was determined, as shown in Figure 2. The flight plan was generated automatically using DJI GS RTK (SZ DJI Technology) to cover the analysis region of each field with an adequate margin. The flight height was 25 m, the forward overlapping rate was 80%, and the side-overlapping rate was 60%. For the flight in Field 1, the camera angle (angles of a camera’s forward direction from a horizontal plane) was –90°, and RTK was not used. For the flight in Field 2, the camera angle was set to –60° or –90° as shown in Figure 3, and RTK was switched on or off. Therefore, four flight conditions were applied. The details of UAV image acquisition are shown in Supplementary Table 1 (Field 1) and Supplementary Table 2 (Field 2).

In Field 1, three flight repetitions were conducted at each growth stage. For Field 2, image acquisition was conducted in three stages, namely, the pre-germination stage (for obtaining off-season data of method M1), the vegetative stage, and the reproductive stage. At each stage, 12 flights (four conditions with three flight repetitions) were arranged using a randomized block design.

The SfM process was conducted using the Agisoft Metashape Professional 1.7.3 (Agisoft LLC, St. Petersburg, Russia). Three-dimensional point clouds were generated from the image sets, and orthomosaic images and DSMs were constructed. The parameters selected for the process are listed in Table 2. From each image set of Field 2, another Metashape project file was created for the GCP-corrected analysis. In the project file, markers were set at the locations of nine GCPs, the coordinates of GCPs by the ground survey were input, and the SfM process was conducted in a similar manner. The conditions for the SfM products are summarized in Supplementary Table 3.

**TABLE 2:** Parameters of the SfM process.

Process	Parameter	Setting
Aligning photos	Accuracy	High
	Generic preselection	Yes
	Key point limit	40,000
	Tie point limit	4,000
Building dense point cloud	Quality	High
	Depth filtering modes	Mild
Building digital elevation model	Source data	Dense cloud
	Interpolation	Enabled
Building orthomosaic	Surface	Digital elevation model
	Blending mode	Mosaic
	Hole filling	Enabled

### Methods for Analysis of Digital Surface Model

Each analysis row in each field was divided into three blocks. Using QGIS Desktop 3.16.8, one polygon enclosing each field (Field 1 or 2) and polygon enclosing blocks were created on each orthomosaic image and written to a shapefile (the locations of polygons are shown in Supplementary Figure 1). Regions of interest (ROIs) in rows (inner ROIs, for all methods) and around the field (outer ROIs, for method M3) were determined using the shapefile. For the inner ROIs (red rectangles in Figure 2), the coordinates were calculated with the locations of the plants measured in the blocks. For outer ROIs (blue rectangles), coordinates of 180 rectangular areas with a size of approximately 1 × 0.5 m (on the corner: 0.5 × 0.5 m) enclosing each field were calculated. The coordinates were saved as CSV files. In Figure 2, the locations of the ROIs are drawn on an orthomosaic according to the coordinates used for visualization.

For method M1 (only Field 2), an off-season DSM was used as the DTM, which was subtracted from the on-season DSM. For each on-season DSM, an off-season DSM under the same conditions and repetition was applied; thus, the same number of CHMs (24 CHMs for Field 2) were obtained.

**FIGURE 4:**
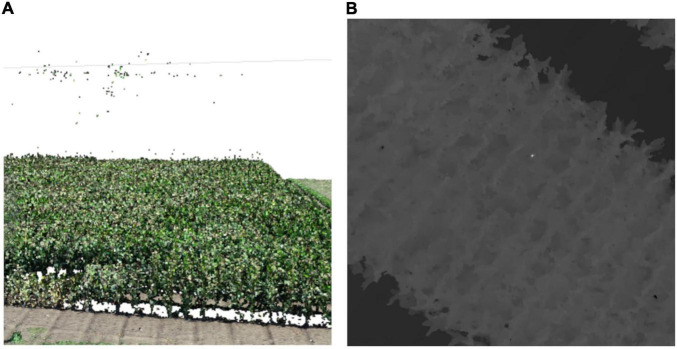
An example of noises in point clouds. **(A)** A point cloud with noises (some points floating over plants). **(B)** A DSM with noises (an extremely high area near the center). The DSM is shown in grayscale; when the pixel is white, the altitude is high.

The 90th–99th percentiles have often been applied as representative values of PH (Holman et al., 2016; Malambo et al., 2018; Tirado et al., 2020), as they express height (altitude of the highest point) better than a mean and are subjected to less noise than a maximum. In this study, the size of an inner ROI was approximately 2,500 pixels, and noises that were blobs of adjacent 30 pixels or smaller were observed on a DSM (Figure 4). Therefore, to exclude noise less than 2% of the ROI (50 pixels), the 98th percentile was used in this study. For the CHMs, the 98th percentile from the inner ROIs was calculated as *PH*_SfM_.

For method M2 (only Field 2), the 2nd and 98th percentiles from the inner ROIs were calculated as the altitude of the ground and plant apex, respectively. These percentiles were applied for the same reason mentioned above. The difference between the 2nd and 98th percentiles was obtained as *PH*_SfM_.

For method M3 (Fields 1 and 2), a DTM was obtained by polynomial fitting with the coordinates of the terrain around the field on an on-season DSM. The median altitude was calculated from each outer ROI as the *z*-coordinate of the area, and the center of gravity of the rectangle was calculated as the *x* and *y*-coordinates. A total of 180 points (*x*, *y*, *z*) were fitted to an *n*-dimensional polynomial surface (1) using the least squares method.


(1) $$z=\sum_{k=0}^{n}\sum_{i=0}^{k}a_{k\,i}\,x^{k-i}\,y^{i}$$

where *n* is the dimension of the polynomial surface, *k* and *i* are the indices for summation, and *a*_*ki*_ is the parameter to be estimated. This polynomial surface was used as the DTM. This DTM was subtracted from the original DSM to obtain a CHM. On the CHMs, the 98th percentiles from the inner ROIs were calculated as *PH*_SfM_ (PH obtained from UAV-SfM analysis).

The dimension of the polynomial (*n*) was set to 0–4 for data from Field 1. The dimension that achieved the strongest correlation between the measured PH (*PH*_measured_) and *PH*_SfM_ in Field 1 was applied for a comparative study of Field 2.

The analysis in this and the following sections were conducted using Python 3.6.8 and QGIS.

### Correlation Analysis and Cross-Validation of Regression Models

The Pearson correlation coefficient (*r*) between *PH*_measured_ and *PH*_SfM_ in each dataset and the bias (*PH*_*SfM*_−*PH*_*measured*_) were calculated. For each condition, the three coefficients obtained from the repetitions were averaged.

Cross-validation of the linear regression models to predict *PH*_measured_ from *PH*_SfM_ was conducted using data from Field 2. As the SfM point clouds from different flights could deviate from each other, validation with different target data on a different flight’s point cloud was needed (“different-targets-and-different-flight” validation) to ensure that a regression model from one flight can be applied to as training data to unknown data. The 168 data points from Field 2 were divided into three groups (the grouping in Field 2 is shown in Figure 2). A linear regression model (2) was fitted with the least-squares method using data from two groups (112 training data points).


(2) $$P\,H_{m\,e\,a\,s\,u\,r\,e\,d}=a\times P\,H_{\mathrm{SfM}}+b$$

where *a* and *b* are the parameters to be estimated (*a*: slope and *b*: intercept, respectively). A total of 56 data points from different flight repetition groups were used for validation. There were six flight repetitions and three groups for training and validation; thus, 18 sets of validations were conducted for each condition (all sets of flight repetitions and sample groups for cross-validation are shown in Supplementary Table 4). The coefficient of determination (*R*^2^), mean absolute error (MAE), root mean squared error (RMSE), and mean absolute percentage error (MAPE) were calculated using the validation data (equations of these evaluation metrics are shown in Supplementary Table 5).

## Results

### Consideration of Method M3

A summary of the measured PH (*PH*_measured_) is provided in Table 3. The range of the measured PH was 0.632–1.190 m in the vegetative stage and 2.18–3.14 m in the reproductive stage. The standard deviations in Fields 1 and 2 were 0.075 and 0.102 m in the vegetative stage and 0.126 and 0.181 m in the reproductive stage, respectively. These results indicate high variability in PH in the fields.

**TABLE 3:** Descriptive statistics of measured PH.

	Vegetative stage		Reproductive stage	
		
	Field 1	Field 2	Field 1	Field 2
Date of measurement	28-Jun	30-Jun	6-Aug	3-Sep
Number of samples	84	168	84	168
Mean (m)	0.809	0.898	2.73	2.68
Minimum (m)	0.634	0.632	2.47	2.18
Maximum (m)	0.982	1.190	3.05	3.14
Standard deviation (m)	0.075	0.102	0.126	0.181

**FIGURE 5:**
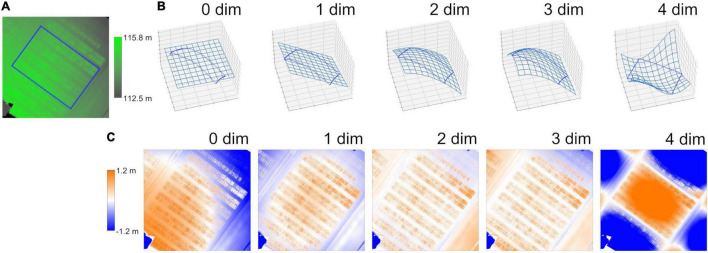
An example of the process involved in method M3. **(A)** A digital surface model (DSM) of Field 1. Blue rectangles show ROIs around the field (outer ROIs). **(B)** Digital terrain models (DTMs) fitted to polynomial surfaces. Blue points show coordinates of the outer ROIs and meshes show the fitted DTMs. “*n* dim” shows the dimension of the polynomial surface. **(C)** Crop height models (CHMs) calculated as the difference between DSM and DTMs. These figures were created with the dataset of rep. 1 on Field 1 in the vegetative stage.

The DSM of Field 1 shows ground inclination, with the northeast being lower and the southwest being higher (Figure 5). The CHM calculated from the 0 dim (flat) DTM left the inclination, as the 0 dim DTM cannot model such a tilted plane. The CHM calculated from the 1 dim (plane) DTM did not leave the inclination but left the central bulge. The 2 and 3 dim DTMs fitted better to the true terrain. The CHMs from the 2 and 3 dim DTMs left neither the inclination nor bulge inside the ground ROIs. The 4 dim DTM, however, overfitted the sample points of the ground, and thus, the CHM from the DTM was strongly distorted.

The mean correlation coefficients (*r*) between *PH*_measured_ and *PH*_SfM_ on the CHMs of Field 1 from the three flight repetitions are summarized in Table 4. The correlation was stronger with the 3 dim DTM in both growth stages. Thus, the 3 dim DTM was applied to method M3 in validation with Field 2.

**TABLE 4:** Correlation coefficients (*r*) between *PH*_measured_ and *PH*_SfM_ on the CSMs of Field 1.

Dimension of polynomial surface	0 dim	1 dim	2 dim	3 dim	4 dim
Vegetative stage	0.469	0.776	0.832	**0.839**	0.153
Reproductive stage	0.346	0.816	0.866	**0.873**	0.272

*Each value is the mean of 3 flight repetitions. 3 dim (bolded) was highest in the correlation.*

### Comparative Correlation Analysis Between *PH*_measured_ and *PH*_SfM_ on All Conditions

*PH*_SfM_ of Field 2 was calculated for 24 conditions that differed in the four parameters, namely, camera angles, RTK, GCPs, and methods for obtaining GA (method M1: using off-season DSM, M2: extracting altitude of the soil surface, and M3: fitting coordinates of the terrain around the field to a polynomial surface). The correlation coefficients (*r*) between *PH*_measured_ and *PH*_SfM_ were compared at each growth stage (vegetative stage, Table 5; reproductive stage, Table 6).

**TABLE 5:** Correlation analysis between *PH*_measured_ and *PH*_SfM_ in the vegetative stage of Field 2.

	Camera angle	RTK	GCP	Method*	*r* ^†^	Bias (m)^†^
1	−60°	+	+	M3	**0.914**	−0.078
2	−60°	+	−	M3	**0.913**	−0.080
3	−60°	−	+	M3	**0.906**	−0.087
4	−60°	−	−	M3	**0.906**	−0.087
5	−60°	+	+	M1	**0.903**	−0.103
6	−60°	+	−	M1	**0.903**	−0.135
7	−60°	−	+	M1	**0.896**	−0.115
8	−90°	+	+	M3	**0.894**	−0.055
9	−90°	+	−	M3	**0.891**	−0.070
10	−90°	−	+	M3	**0.888**	−0.058
11	−90°	+	+	M2	**0.886**	−0.035
12	−90°	−	−	M3	**0.885**	−0.077
13	−90°	+	+	M1	**0.885**	−0.089
14	−90°	+	−	M1	**0.874**	0.842
15	−90°	−	+	M2	**0.874**	−0.040
16	−90°	−	+	M1	**0.874**	−0.091
17	−90°	−	−	M2	**0.868**	−0.058
18	−60°	−	−	M1	**0.856**	−1.023
19	−90°	+	−	M2	**0.850**	−0.052
20	−90°	−	−	M1	**0.810**	0.334
21	−60°	−	−	M2	**0.539**	−0.096
22	−60°	−	+	M2	**0.494**	−0.095
23	−60°	+	−	M2	**0.333**	−0.111
24	−60°	+	+	M2	**0.330**	−0.106

*Method for obtaining ground altitude (GA); M1, using off-season DSM; M2, extracting altitude of the soil surface; M3: fitting coordinates of the terrain around the field to a polynomial surface.

*^†^r is the correlation coefficient between PH_measured_ and PH_SfM_, and bias is the mean of the difference between PH_measured_ and PH_SfM_ (PH_SfM_−PH_measured_). Each value of r and bias is the mean of three flight repetitions. The rows are sorted by r (bolded) in a descending order.*

**TABLE 6:** Correlation analysis between *PH*_measured_ and *PH*_SfM_ in the reproductive stage of Field 2.

	Camera angle	RTK	GCP	Method*	*r* ^†^	Bias (m)^†^
1	−60°	+	+	M1	**0.907**	−0.262
2	−60°	+	−	M1	**0.906**	−0.306
3	−60°	+	+	M3	**0.899**	−0.227
4	−60°	+	−	M3	**0.899**	−0.233
5	−60°	−	+	M1	**0.894**	−0.267
6	−60°	−	+	M3	**0.886**	−0.232
7	−60°	−	−	M3	**0.883**	−0.236
8	−90°	+	−	M1	**0.869**	0.790
9	−90°	+	+	M1	**0.863**	−0.210
10	−60°	−	−	M1	**0.862**	−1.181
11	−90°	+	−	M3	**0.846**	−0.212
12	−90°	+	+	M3	**0.845**	−0.166
13	−90°	−	+	M1	**0.843**	−0.208
14	−90°	−	+	M3	**0.829**	−0.167
15	−90°	−	−	M3	**0.824**	−0.213
16	−90°	−	−	M1	**0.809**	0.045
17	−90°	+	−	M2	**0.327**	−1.745
18	−90°	+	+	M2	**0.316**	−1.736
19	−60°	+	−	M2	**0.281**	−1.821
20	−60°	−	−	M2	**0.259**	−1.836
21	−60°	+	+	M2	**0.257**	−1.790
22	−90°	−	−	M2	**0.243**	−1.783
23	−90°	−	+	M2	**0.229**	−1.769
24	−60°	−	+	M2	**0.208**	−1.828

*Method for obtaining ground altitude (GA); M1, using off-season DSM; M2, extracting altitude of the soil surface; M3, fitting coordinates of the terrain around the field to a polynomial surface.

*^†^r is the correlation coefficient between PH_measured_ and PH_SfM_, and bias is the mean of the difference between PH_measured_ and PH_SfM_ (PH_SfM_−PH_measured_). Each value of r and bias is the mean of three flight repetitions. The rows are sorted by r (bolded) in a descending order.*

In the vegetative stage (Table 5), the correlation was stronger when a camera angle of −60° (diagonal) and method M3 were applied, even without RTK or GCPs. Using method M1, the correlation was stronger when RTK or GCPs were present. With method M2, although the correlation was strong at a −90° (nadir) camera angle, it was weak at −60°. The bias (*PH*_*SfM*_−*PH*_*measured*_) was negative (−0.07 to −0.09 m) for the highest four conditions in correlation coefficients. *PH*_SfM_ tended to be lower than *PH*_measured_.

In the reproductive stage (Table 6), for both methods M1 and M3, the correlation was strong with −60° camera angle and RTK. When RTK and GCPs were not applied, the correlation was stronger using method M3. In the M2 method, the correlation was weak. *PH*_SfM_ tended to be lower than *PH*_measured_ during the reproductive stage (bias = approx. −0.2 to −0.3 m under higher correlation coefficient conditions).

### Cross-Validation of Plant Height Regression Models

A linear regression model was necessary for PH prediction with UAV-SfM because *PH*_SfM_ tended to be lower than *PH*_measured_. Simple regression models for PH prediction were trained and the “different-targets-and-different-flight” cross-validation was conducted under all conditions (vegetative stage: Table 7, reproductive stage: Table 8). In the reproductive stage, method M2 was omitted because the correlation between *PH*_measured_ and *PH*_SfM_ was weak (Table 6).

**TABLE 7:** Evaluation metrics on cross-validation of PH regression models in the vegetative stage of Field 2.

	Camera angle	RTK	GCP	method*		$R_{\text{train}}^{2}†$	$R_{\text{val}}^{2}†$	MAE (m)^†^	RMSE (m)^†^	MAPE^†^
1	−60°	−	+	M3		0.820	**0.799**	0.036	0.044	4.08
2	−60°	−	−	M3	(LC)^‡^	0.820	**0.794**	0.036	0.045	4.14
3	−60°	−	+	M1		0.801	**0.786**	0.037	0.046	4.17
4	−60°	+	+	M3		0.835	**0.770**	0.038	0.047	4.36
5	−60°	+	−	M3		0.832	**0.769**	0.038	0.047	4.34
6	−60°	+	+	M1	(HC)^‡^	0.815	**0.766**	0.039	0.048	4.36
7	−90°	−	+	M3		0.786	**0.758**	0.038	0.049	4.38
8	−90°	−	−	M3		0.782	**0.756**	0.039	0.049	4.43
9	−90°	+	−	M3		0.793	**0.745**	0.039	0.049	4.42
10	−90°	+	+	M3		0.799	**0.737**	0.040	0.050	4.56
11	−90°	+	+	M2		0.783	**0.729**	0.041	0.051	4.63
12	−90°	+	+	M1		0.781	**0.728**	0.041	0.051	4.61
13	−90°	−	+	M2		0.762	**0.722**	0.041	0.052	4.61
14	−90°	−	+	M1		0.762	**0.720**	0.042	0.052	4.69
15	−90°	−	−	M2		0.753	**0.703**	0.042	0.053	4.74
16	−90°	+	−	M2		0.727	**0.669**	0.043	0.057	4.83
17	−60°	+	−	M1		0.814	**0.401**	0.063	0.073	7.14
18	−60°	−	+	M2		0.253	**0.200**	0.065	0.090	7.32
19	−60°	−	−	M2		0.317	**0.167**	0.063	0.091	7.04
20	−60°	+	−	M2		0.146	−**0.045**	0.077	0.102	8.65
21	−60°	+	+	M2		0.142	−**0.066**	0.077	0.103	8.71
22	−60°	−	−	M1		0.731	−**40.07**	0.582	0.585	65.78
23	−90°	+	−	M1		0.764	−**74.45**	0.774	0.776	87.23
24	−90°	−	−	M1		0.658	−**99.50**	0.791	0.796	88.84

*Method for obtaining ground altitude (GA); M1, using off-season DSM; M2, extracting altitude of the soil surface; M3, fitting coordinates of the terrain around the field to a polynomial surface.

*^†^R2t⁢r⁢a⁢i⁢n and R2v⁢a⁢l are the coefficients of determination for the training and validation datasets, respectively. MAE is the mean absolute error, RMSE is the root mean squared error, and MAPE is the mean absolute percentage error of the validation data. Each value represents the mean of the 18 validation cases.*

*^‡^LC means “Low-cost case” (camera angle: −60°, RTK: unused [−], GCPs: unused [−], method: M3), and HC means “Highest-cost case” (camera angle: −60°, RTK: used [+], GCPs: used [+], method: M1).*

*The rows are sorted by R^2^_val_ (bolded) in a descending order.*

**TABLE 8:** Evaluation metrics on cross-validation of PH regression models in the reproductive stage of Field 2.

	Camera angle	RTK	GCP	Method*		$R_{\text{train}}^{2}†$	$R_{\text{val}}^{2}†$	MAE (m)^†^	RMSE (m)^†^	MAPE^†^
1	−60°	+	+	M1	(HC)^‡^	0.821	**0.803**	0.063	0.078	2.36
2	−60°	+	+	M3		0.810	**0.791**	0.066	0.081	2.47
3	−60°	+	−	M3		0.808	**0.781**	0.067	0.083	2.52
4	−60°	−	+	M1		0.799	**0.771**	0.068	0.085	2.55
5	−60°	−	+	M3		0.785	**0.753**	0.071	0.089	2.67
6	−60°	−	−	M3	(LC)^‡^	0.782	**0.749**	0.072	0.089	2.70
7	−90°	+	+	M1		0.745	**0.640**	0.086	0.105	3.23
8	−90°	+	+	M3		0.718	**0.601**	0.091	0.111	3.40
9	−90°	−	+	M1		0.712	**0.597**	0.089	0.112	3.32
10	−90°	+	−	M3		0.721	**0.592**	0.093	0.113	3.47
11	−90°	−	−	M3		0.680	**0.576**	0.093	0.115	3.48
12	−90°	−	+	M3		0.691	**0.562**	0.093	0.116	3.48
13	−60°	+	−	M1		0.821	**0.548**	0.097	0.115	3.64
14	−60°	−	−	M1		0.746	−**14.240**	0.637	0.644	23.83
15	−90°	+	−	M1		0.756	−**15.510**	0.608	0.619	22.74
16	−90°	−	−	M1		0.653	−**49.841**	1.115	1.122	41.62

*Method for obtaining ground altitude (GA); M1, using off-season DSM; M3, fitting coordinates of the terrain around the field to a polynomial surface.

*^†^R2t⁢r⁢a⁢i⁢n and R2v⁢a⁢l are the coefficients of determination for the training and validation datasets, respectively. MAE is the mean absolute error, RMSE is the root mean squared error, and MAPE is the mean absolute percentage error of the validation data. Each value represents the mean of the 18 validation cases.*

*^‡^LC means “Low-cost case” (camera angle: −60°, RTK: unused [−], GCPs: unused [−], method: M3), and HC means “Highest-cost case” (camera angle: −60°, RTK: used [+], GCPs: used [+], method: M1).*

*The rows are sorted by R^2^_val_ (bolded) in a descending order.*

In the vegetative stage (Table 7), the coefficient of determination of the validation data (R2val) was high when the −60° camera angle and method M3 were applied, as was the correlation coefficient between *PH*_measured_ and *PH*_SfM_ (Table 5). Even in the “Low-cost case (LC)” (camera angle: −60°, RTK: unused [−], GCPs: unused [−], method: M3), which can be conducted with minimum equipment and without an extra flight, the regression model showed a high predictive performance (R2val = 0.794, MAE = 0.036 m). In the “Highest-cost case (HC)” (camera angle: −60°, RTK: used [+], GCPs: used [+], method: M1), which seems to achieve high-precision sensing with method M1, the R2val was 0.766, which was lower than that of LC. With method M1, R2val was high only when GCPs were used.

The predictive performance of HC was highest in the reproductive stage (Table 8) (R2val = 0.803, MAE = 0.063 m). R2val was also high when the camera angle was −60° and method M3 was applied, including LC (R2val = 0.749, MAE = 0.072 m). With method M1, the predictive performance was low when GCPs were not used, similar to the vegetative stage. Although the overall mean absolute errors in the validation data (MAEs) in the reproductive stage were larger than those in the vegetative stage, the mean absolute percentage errors (MAPEs) in the reproductive stage were smaller (MAPE = 2–3% in the six highest conditions in R2val).

**FIGURE 6:**
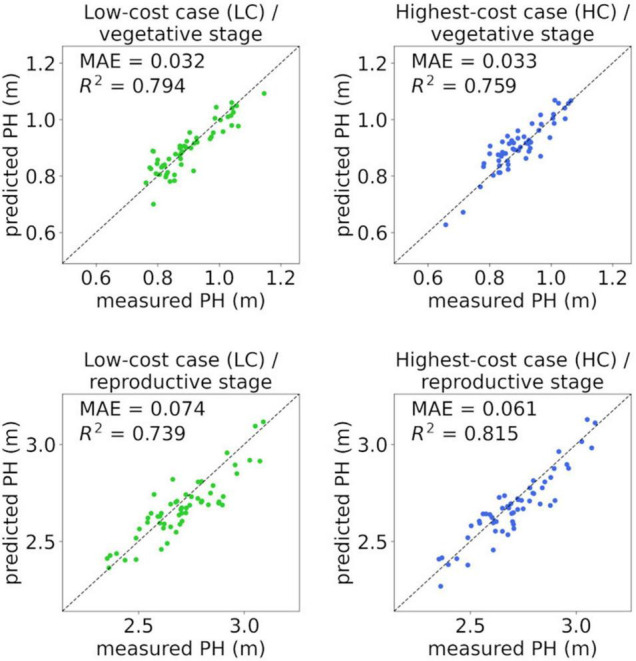
Scatterplots between measured PH (*PH*_measured_) and PH predicted by the linear regression model from *PH*_SfM_ on validation data. “Low-cost case (LC)” is the condition with camera angle: −60°, RTK: unused [−], GCPs: unused [−], method: M3; and “Highest-cost case (HC)” is the condition with camera angle: −60°, RTK: used [+], GCPs: used [+], method: M1. In each set of analysis conditions (LC or HC) and stage (vegetative or reproductive), the nearest to the mean in R2val was selected from 18 validation cases.

Examples of cross-validation, that is, scatterplots between the measured PH (*PH*_measured_) and PH predicted by the regression model from *PH*_SfM_ on the validation data, are shown in Figure 6. From 18 validation cases for each condition, the average case (nearest to the mean in R2val) was selected for the figure.

## Discussion

**FIGURE 7:**
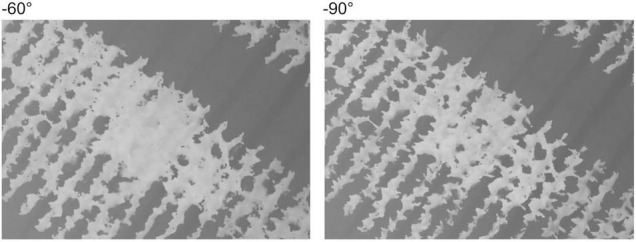
A comparison of DSMs with −60° and −90° for the camera angle. The DSMs are shown in grayscale; when the pixel is white, the altitude is high.

The correlation between *PH*_measured_ and *PH*_SfM_ was stronger with a −60° camera angle than with −90°, except for method M2 (Tables 5, 6). This tendency for a strong correlation of −60° was observed even when GCPs were used. Therefore, the diagonal camera angle could both suppress the “doming” effect and grasp the 3D structures of the plants well with a lateral view. However, in the vegetative stage using method M2, the soil surface in some plots was difficult to image at a diagonal camera angle, and the low accuracy of the GA appeared to result in a weak correlation. In some examples, a DSM with a −60° camera angle had wider plant areas than a DSM with a −90° camera angle and a hidden soil surface (Figure 7). In the reproductive stage, the inner ROI was mostly covered with plants, and thus, M2 was difficult to apply with both −60° and −90° camera angles.

The R2val ranks of the regression models differed from those of the correlation coefficients (*r*) between *PH*_measured_ and *PH*_SfM_ (Tables 5–8). For example, in the vegetative stage, the condition with camera angle: −60°, RTK: used [+], GCPs: unused [−], and method: M1 scored high correlation coefficients (*r* = 0.903, rank = 6; Table 5) and thus high goodness of fit for the training data (R2train = 0.814; Table 7). However, the regression models had low predictive performance on “different-targets-and-different-flight” validation data (R2val = 0.401, rank = 17; Table 7). Although a strong correlation in one flight leads to high goodness of fit for the training data, the model showed low predictive performance on unknown data from different flights.

The predictive performance on unknown data was higher with the M1 and GCP methods or with method M3 (Tables 7, 8). With method M1, GCPs seemed to prevent the deviation between 3D models from different flights and contributed to the high predictive performance. For method M3, the predictive performance was not affected by such deviation, even without GCPs. The ground surface was determined for each on-season flight by using method M3. In this process, the effect of the overall deviation was reduced.

The contribution of RTK positioning to the precision of PH monitoring was restricted in this study, although only small effects were observed. With RTK used [+], GCPs unused [−], and method M1 applied, the R2val was lower despite some improvement by a diagonal (−60°) camera angle (Tables 7, 8). Although RTK positioning installed on UAVs enables centimeter-level precision on a DSM (Forlani et al., 2018), the differences in PH were also at the centimeter level. Moreover, SfM photogrammetry based on RTK positioning has larger vertical errors than horizontal errors (Forlani et al., 2018; Štroner et al., 2020). A previous study on a paddy field under similar conditions of UAV image acquisition reported a 0.031 m vertical coordinate error (MAE) with a −60° camera angle and 2.10 m with a −90° camera angle on UAV-SfM point clouds with RTK without GCPs (Fujiwara et al., in press). A slight vertical deviation of point clouds with RTK positioning may cause low predictive performance for different flight data. For UAV-SfM reproducibility between different flights, GCPs seem to be more reliable than RTK.

In this study, the regression models in the two growth stages were trained separately. In contrast, a common model across growth stages scored a high coefficient of determination (*R*^2^) in several studies (Madec et al., 2017; Tirado et al., 2020; Lu et al., 2022). Considering that data from multiple growth stages have high variance, the proportion of the variation explained by the model could be large. That is, when the data obtained from an early stage (e.g., PH = 0.5–1 m) and from a later stage (e.g., PH = 2–3 m) are mixed and fitted to a model, a high coefficient of determination is expected. However, errors such as MAE and RMSE may be larger than those specific to the growth stage. Stage-specific models are important for simultaneous evaluation. In this study, PH prediction of centimeter-level accuracy was made possible by separating the models from the vegetative and reproductive stages. Strategies should be selected by considering the target and accuracy.

With method M3, the coordinates of the terrain are extracted only around a field, and thus, this method is applicable to a field covered with plants. In this study, although the trial fields had passages without plants (Figure 2), these passages were not used as GA. This was because of the assumption of production fields without such a passage. It was shown that method M3 worked when the inside of the field was covered with plants.

In this study, all outer ROIs were used for polynomial fitting because bare soil was always visible, that is, weeds were few. When such bare soil areas around a field are unavailable, it may be better to eliminate some outer ROI areas. For a field completely covered with plants without any margin, applying method M3 would be difficult. In such a situation, method M1 with RTK, GCP, or both may be more suitable.

The shapefiles for ROIs were created on each orthomosaic in this study; thus, the horizontal deviation of the 3D models did not affect the predictive performance. However, when common ROIs in a field are used for different flights, such horizontal deviations can cause errors. To reduce the cost of creating ROIs on time-series datasets, high-precision positioning with GCPs or RTK could be beneficial, regardless of the method used to obtain GA.

Three-dimensional structural analysis with UAV-SfM is applicable to PH monitoring, yield prediction (Bendig et al., 2014; Li et al., 2016; Roth and Streit, 2018; Jiang et al., 2019; Karunaratne et al., 2020), and lodging detection (Chu et al., 2017; Yang et al., 2017). Moreover, PH data from UAV-SfM, such as the mean, percentiles, and coefficient of variation, can be combined with RGB and multispectral data for crop-monitoring systems (Li et al., 2016; Jiang et al., 2019; Karunaratne et al., 2020). The PH obtained using a high-precision and low-cost method is the basis for advanced demonstrations. The UAV-SfM methods demonstrated in this study can be applied to various targets and analytical strategies.

In this study, to evaluate the predictive performance of unknown data from another flight, a “different-targets-and-different-flight” cross-validation was conducted. It was suggested that method M1 with GCPs and method M3 could build regression models with the goodness of fit to unknown data. Particularly, with method M3, the predictive performance was high on “LC” without the use of GCPs or RTK. Therefore, this could work as a high-precision and low-cost method for general analysis based on UAV-SfM. Three-dimensional structural analysis using this method may prove useful for remote sensing of production fields.

## Data Availability Statement

The original contributions presented in the study are included in the article/Supplementary Material, further inquiries can be directed to the corresponding author/s.

## Author Contributions

RF, TK, HS, and YA designed this study and performed experiments. RF has contributed new analytical tools and analyzed the data. RF and YA wrote the manuscript. All authors have contributed to the manuscript and approved the submitted version.

## Conflict of Interest

The authors declare that the research was conducted in the absence of any commercial or financial relationships that could be construed as a potential conflict of interest.

## Publisher’s Note

All claims expressed in this article are solely those of the authors and do not necessarily represent those of their affiliated organizations, or those of the publisher, the editors and the reviewers. Any product that may be evaluated in this article, or claim that may be made by its manufacturer, is not guaranteed or endorsed by the publisher.

## Abbreviations

CHM: crop height model
DSM: digital surface model
DTM: digital terrain model
GA: ground altitude
GCP: ground control point
GNSS: global navigation satellite system
HC: highest-cost case
LC: low-cost case
LiDAR: light detection and ranging
MAE: mean absolute error
MAPE: mean absolute percentage error
PH: plant height
RMSE: root mean squared error
ROI: region of interest
RTK: real-time kinematic
SfM: structure from motion
UAV: unmanned aerial vehicle.
