# Supplementary material for: Comparison of Remote Sensing Methods for Plant Heights in Agricultural Fields Using Unmanned Aerial Vehicle-Based Structure From Motion
Source: Front Plant Sci. 2022 Jun 24;13:886804. doi: 10.3389/fpls.2022.886804 (PMC9263916; doi:10.3389/fpls.2022.886804)
Supplement: Supplementary file 1 [file Data_Sheet_1.docx]

**Supplementary Material**

**Supplementary Tables**

Supplementary Table S1. The detail of UAV image acquisition on Field 1.

| Stage | Camera  angle | RTK | Repetition | Date | Time | Number of images |
| --- | --- | --- | --- | --- | --- | --- |
| Vegetative stage | –90° | – | 1 | 2021/6/28 | 09:44 - 09:52 | 176 |
|  | –90° | – | 2 | 2021/6/28 | 10:07 - 10:15 | 176 |
|  | –90° | – | 3 | 2021/6/28 | 10:43 - 10:51 | 176 |
| Reproductive stage | –90° | – | 1 | 2021/8/4 | 09:26 - 09:33 | 176 |
|  | –90° | – | 2 | 2021/8/4 | 09:46 - 09:54 | 176 |
|  | –90° | – | 3 | 2021/8/4 | 10:34 - 10:42 | 176 |

The date and time are based on Japan Standard Time (JST = UT + 9 hours).

Supplementary Table S2. The detail of UAV image acquisition on Field 2.

| Stage | Camera  angle | RTK | Repetition | Date | Time | Number of images |
| --- | --- | --- | --- | --- | --- | --- |
| Pre-germination | –60° | – | 1 | 2021/5/14 | 14:03 - 14:14 | 273 |
|  | –90° | – | 1 | 2021/5/14 | 14:24 - 14:32 | 203 |
|  | –60° | + | 1 | 2021/5/14 | 15:47 - 15:58 | 273 |
|  | –90° | + | 1 | 2021/5/14 | 16:33 - 16:41 | 203 |
|  | –60° | + | 2 | 2021/5/17 | 10:34 - 10:45 | 273 |
|  | –90° | + | 2 | 2021/5/17 | 10:46 - 10:57 | 203 |
|  | –60° | – | 2 | 2021/5/17 | 11:00 - 11:11 | 273 |
|  | –90° | – | 2 | 2021/5/17 | 11:17 - 11:25 | 203 |
|  | –60° | + | 3 | 2021/5/17 | 13:05 - 13:16 | 273 |
|  | –60° | – | 3 | 2021/5/17 | 13:20 - 13:31 | 273 |
|  | –90° | + | 3 | 2021/5/17 | 13:44 - 13:53 | 203 |
|  | –90° | – | 3 | 2021/5/17 | 13:55 - 14:04 | 203 |
| Vegetative stage | –60° | + | 1 | 2021/6/30 | 08:25 - 08:36 | 273 |
|  | –90° | + | 1 | 2021/6/30 | 08:38 - 08:47 | 203 |
|  | –60° | – | 1 | 2021/6/30 | 08:49 - 09:00 | 273 |
|  | –90° | – | 1 | 2021/6/30 | 09:03 - 09:11 | 203 |
|  | –60° | + | 2 | 2021/6/30 | 09:14 - 09:25 | 273 |
|  | –90° | + | 2 | 2021/6/30 | 09:28 - 09:36 | 203 |
|  | –60° | – | 2 | 2021/6/30 | 09:39 - 09:50 | 273 |
|  | –90° | – | 2 | 2021/6/30 | 09:53 - 10:01 | 203 |
|  | –90° | – | 3 | 2021/6/30 | 10:01 - 10:10 | 203 |
|  | –60° | – | 3 | 2021/6/30 | 10:14 - 10:25 | 273 |
|  | –90° | + | 3 | 2021/6/30 | 10:28 - 10:36 | 203 |
|  | –60° | + | 3 | 2021/6/30 | 10:39 - 10:50 | 273 |
| Reproductive stage | –60° | + | 1 | 2021/9/2 | 08:27 - 08:38 | 273 |
|  | –90° | + | 1 | 2021/9/2 | 08:40 - 08:48 | 203 |
|  | –60° | – | 1 | 2021/9/2 | 08:50 - 09:01 | 273 |
|  | –90° | – | 1 | 2021/9/2 | 09:04 - 09:12 | 203 |
|  | –60° | + | 2 | 2021/9/2 | 09:15 - 09:26 | 273 |
|  | –90° | + | 2 | 2021/9/2 | 09:29 - 09:37 | 203 |
|  | –90° | – | 2 | 2021/9/2 | 09:39 - 09:48 | 203 |
|  | –60° | – | 2 | 2021/9/2 | 09:50 - 10:01 | 273 |
|  | –60° | – | 3 | 2021/9/2 | 10:04 - 10:15 | 273 |
|  | –60° | + | 3 | 2021/9/2 | 10:17 - 10:28 | 273 |
|  | –90° | – | 3 | 2021/9/2 | 10:31 - 10:39 | 203 |
|  | –90° | + | 3 | 2021/9/2 | 10:43 - 10:51 | 203 |

The date and time are based on Japan Standard Time (JST = UT + 9 hours).

Supplementary Table S3. Conditions of SfM products (orthomosaic images and DSMs) for Fields 1 and 2.

|  | Stage | Camera  angle | RTK | GCP | Number of repetitions |
| --- | --- | --- | --- | --- | --- |
| Field 1 | Vegetative stage | –90° | – | – | 3 |
|  | Reproductive stage | –90° | – | – | 3 |
| Field 2 | Pre-germination | –60° | + | – | 3 |
|  |  |  |  | + | 3 |
|  |  | –60° | – | – | 3 |
|  |  |  |  | + | 3 |
|  |  | –90° | + | – | 3 |
|  |  |  |  | + | 3 |
|  |  | –90° | – | – | 3 |
|  |  |  |  | + | 3 |
|  | Vegetative stage | –60° | + | – | 3 |
|  |  |  |  | + | 3 |
|  |  | –60° | – | – | 3 |
|  |  |  |  | + | 3 |
|  |  | –90° | + | – | 3 |
|  |  |  |  | + | 3 |
|  |  | –90° | – | – | 3 |
|  |  |  |  | + | 3 |
|  | Reproductive stage | –60° | + | – | 3 |
|  |  |  |  | + | 3 |
|  |  | –60° | – | – | 3 |
|  |  |  |  | + | 3 |
|  |  | –90° | + | – | 3 |
|  |  |  |  | + | 3 |
|  |  | –90° | – | – | 3 |
|  |  |  |  | + | 3 |

Supplementary Table S4. All cross-validation sets of flight repetitions and sample groups on Field 2.

|  | training | | validation | |
| --- | --- | --- | --- | --- |
|  | flight | group | flight | group |
| 1 | Fli. 1 | Group A & B | Fli. 2 | Group C |
| 2 |  |  | Fli. 3 | Group C |
| 3 | Fli. 2 | Group A & B | Fli. 3 | Group C |
| 4 |  |  | Fli. 1 | Group C |
| 5 | Fli. 3 | Group A & B | Fli. 1 | Group C |
| 6 |  |  | Fli. 2 | Group C |
| 7 | Fli. 1 | Group B & C | Fli. 2 | Group A |
| 8 |  |  | Fli. 3 | Group A |
| 9 | Fli. 2 | Group B & C | Fli. 3 | Group A |
| 10 |  |  | Fli. 1 | Group A |
| 11 | Fli. 3 | Group B & C | Fli. 1 | Group A |
| 12 |  |  | Fli. 2 | Group A |
| 13 | Fli. 1 | Group C & A | Fli. 2 | Group B |
| 14 |  |  | Fli. 3 | Group B |
| 15 | Fli. 2 | Group C & A | Fli. 3 | Group B |
| 16 |  |  | Fli. 1 | Group B |
| 17 | Fli. 3 | Group C & A | Fli. 1 | Group B |
| 18 |  |  | Fli. 2 | Group B |

Supplementary Table S5. The equations of evaluation metrics.

| **Metric** | **Equation** |
| --- | --- |
| coefficient of determination | $R^{2}=1-\frac{\sum_{i=1}^{N} {(O_{i}-P_{i})}^{2}}{\sum_{i=1}^{N} {(O_{i}-\bar{O})}^{2}}$ |
| mean absolute error (MAE) | $MAE=\frac{1}{N}\sum_{i=1}^{N} \left\vert P_{i}-O_{i} \right\vert$ |
| root mean squared error (RMSE) | $RMSE=\sqrt{\frac{1}{N}\sum_{i=1}^{N} \left( P_{i}-O_{i} \right)^{2}}$ |
| mean absolute percentage error (MAPE) | $MAPE=\frac{100}{N}\sum_{i=1}^{N} \left\vert\frac{P_{i}-O_{i}}{Oi} \right\vert$ |

$N$ is the number of samples, $O_{i}$ is the observed value, $\bar{O}$ is the mean of the observed values, and $P_{i}$ is the predicted value.

**Supplementary Figure**

**
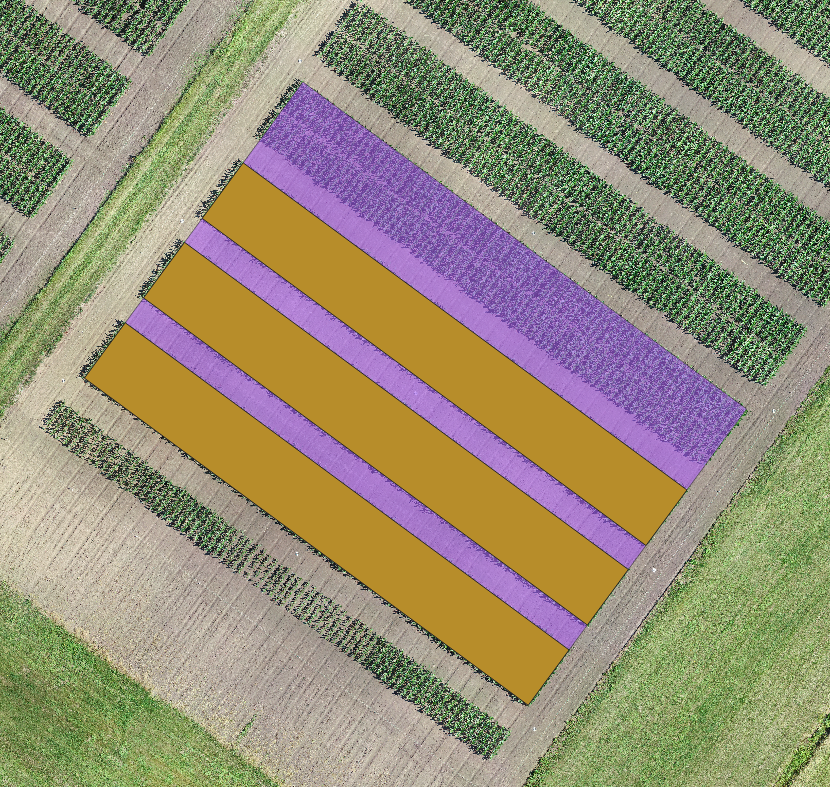
**

Supplementary Figure S1. An example of polygons created using QGIS for setting ROIs. A purple area shows a polygon enclosing a field, and brown areas show polygons enclosing blocks of target.
